# Supplementary material for: h-BN Modification Using Several Hydroxylation and Grafting Methods and Their Incorporation into a PMMA/PA6 Polymer Blend
Source: Nanomaterials (Basel). 2022 Aug 9;12(16):2735. doi: 10.3390/nano12162735 (PMC9414417; doi:10.3390/nano12162735)
Supplement: Supplementary file 1 [file nanomaterials-12-02735-s001.zip › nanomaterials-1789603-supplementary-updated.pdf]

nanomaterials

Supplementary Materials

h-BN Modification Using Several Hydroxylation and Grafting Methods and Their Incorporation into a PMMA/PA6 Polymer Blend

Abdelwahab Boukheit [1], France Chabert [2], Belkacem Otazaghine [1] and Aurélie Taguet [1,*]

[1] Polymers Composites and Hybrids (PCH), IMT Mines Ales, 30319 Ales, France
[2] Laboratoire Génie de Production (LGP), ENIT-INPT University of Toulouse, 65000 Tarbes, France
* Correspondence: aurelie.taguet@mines-ales.fr

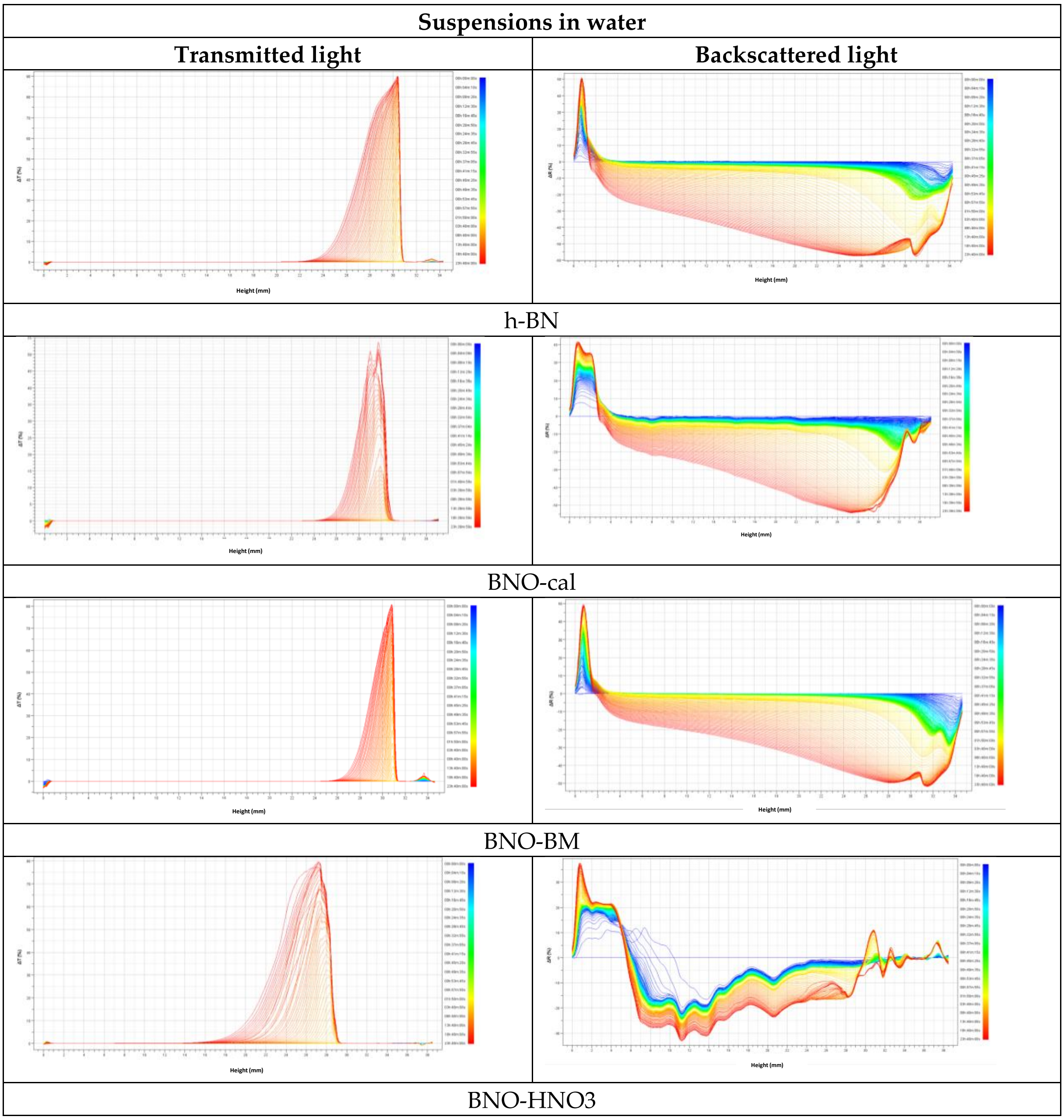

Figure S1. Multiple light scattering results for h-BN and hydroxylated h-BN in water (concentration of 0.5 wt%).

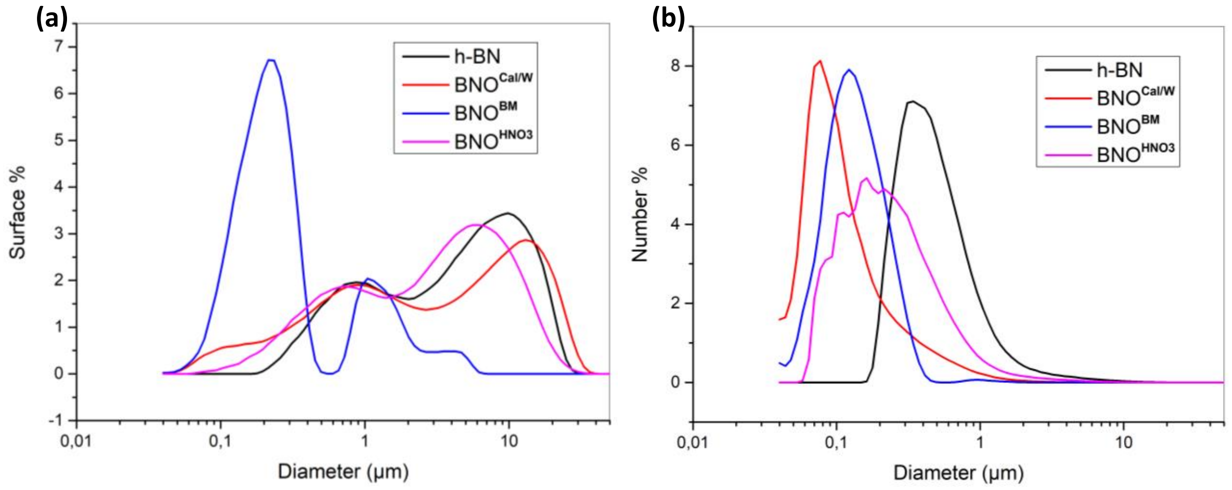

Figure S2. h-BN and hydroxylated BNO laser granulometry in water: (**a**) surface and (**b**) number (concentration of 0.5 wt%).

Since the dispersion of h-BN particles in water was poor, we used the same measurements in isopropanol for its close surface energy with h-BN, as reported by Coleman et al [73]. The h-BN particles demonstrated an average volume size of 1.52 µm (Figure S1), which was in accordance with the SEM images. This proved that h-BN particles are much better dispersed in isopropanol than in water. Another peak was noted at 0.27µm, showing that there were two size populations. However, the peak at 1.5 µm was much more intense even though the SEM images showed that there were more platelets at 0.1-0.5 µm; this was due to the more important volume of the biggest platelets. The $BNO^{Cal/W}$ and BNO^{HNO3} showed similar results, with a slight increase in peak at 2.1 µm. The BNO^{BM} showed a different behavior, where the biggest peak was marked at 12.69 µm, indicating that agglomerates were formed in the sample and that a sonication of more than 1 minute was needed to break those agglomerates. Two other peaks at 1.57 and 0.21 µm were noticed, which confirmed that the ball milling exerted a breakage of particles. Many small pieces were detected, but as their volume was small, their peak was insignificant in comparison with the one at 12 µm. In conclusion, it is safe to say that the results of the SEM images, sedimentation, and granulometry are in agreement.

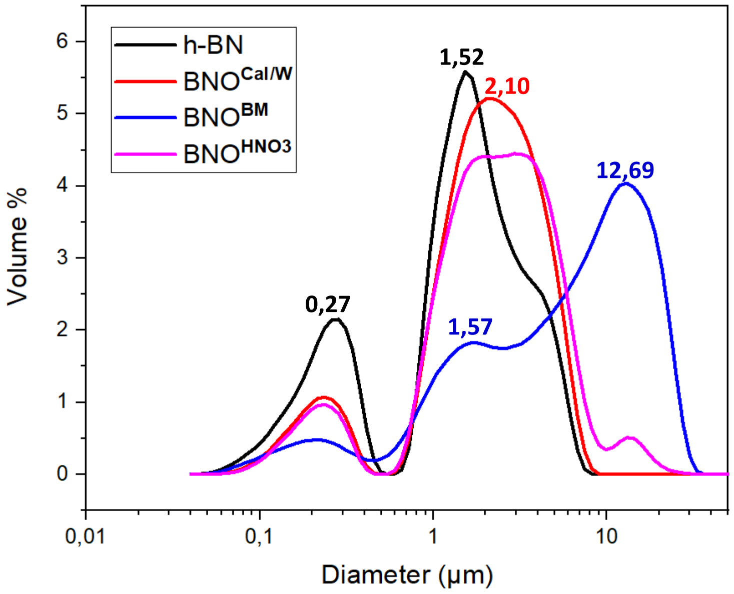

Figure S3. h-BN and hydroxylated BNO granulometry in isopropanol (volume %).

<table>
<tr><th colspan="2">Suspensions in water</th></tr>
<tr><th>Transmitted light</th><th>Backscattered light</th></tr>
<tr><td>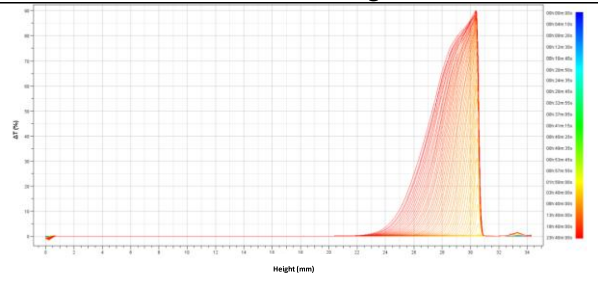</td><td>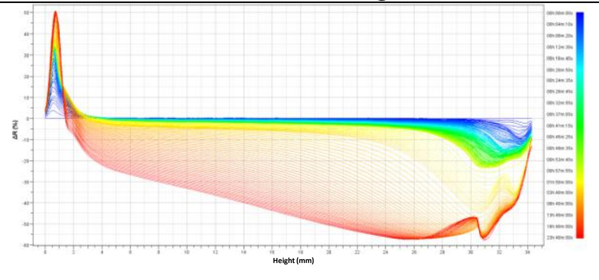</td></tr>
<tr><td colspan="2">h-BN</td></tr>
<tr><td>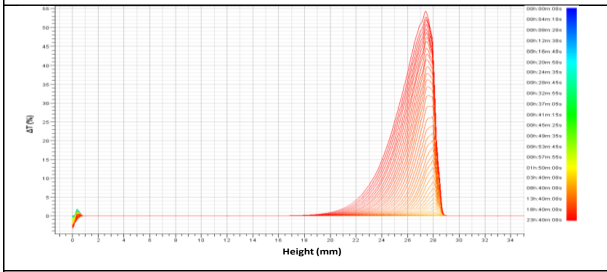</td><td>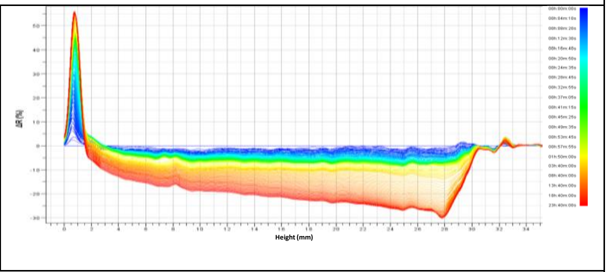</td></tr>
<tr><td colspan="2">BNO-C18</td></tr>
<tr><td>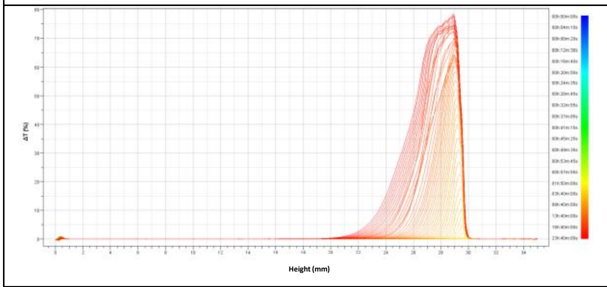</td><td>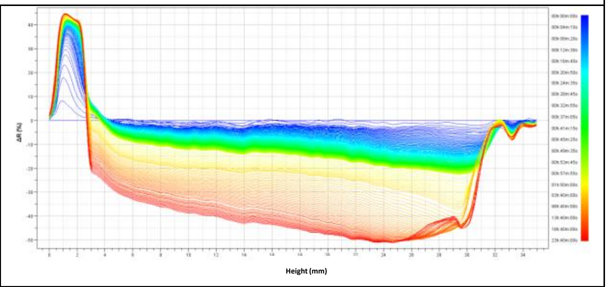</td></tr>
<tr><td colspan="2">BNO-PMMA</td></tr>
<tr><td>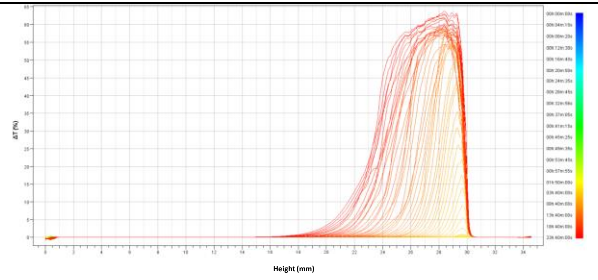</td><td>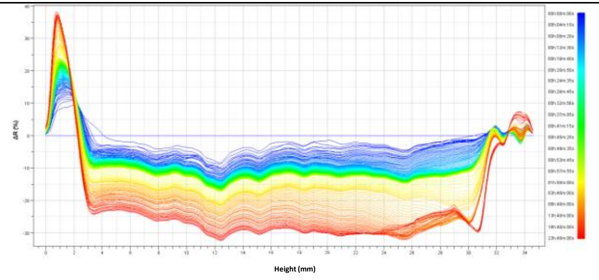</td></tr>
<tr><td colspan="2">BNO-PS</td></tr>
<tr><td>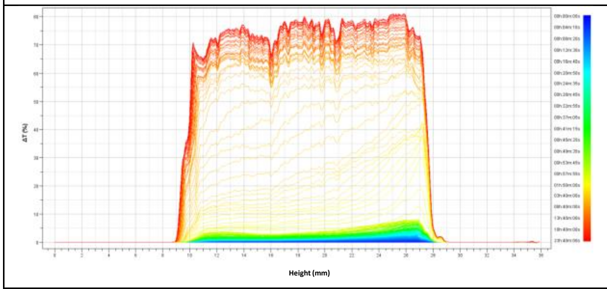</td><td>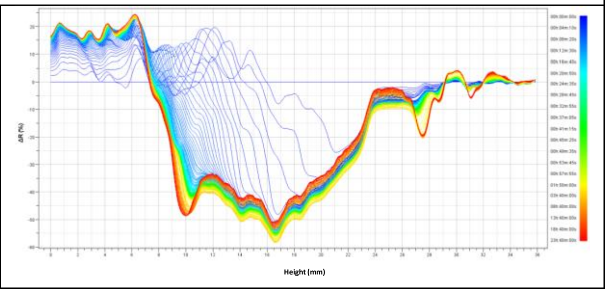</td></tr>
<tr><td colspan="2">BNO-NH2</td></tr>
</table>

Figure S4. Multiple light scattering results for h-BN and silane-grafted h-BN in water.

**Suspensions in toluene**	
Transmitted light	Backscattered light
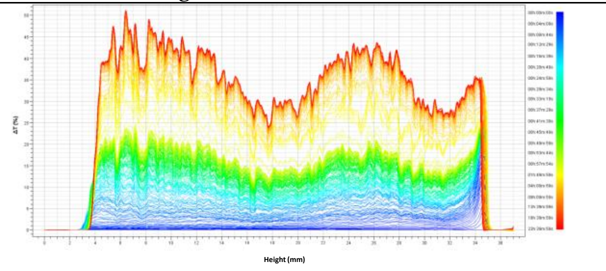	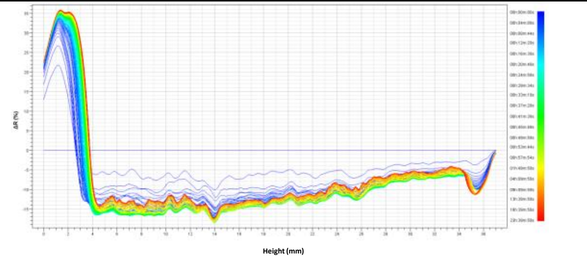
h-BN	
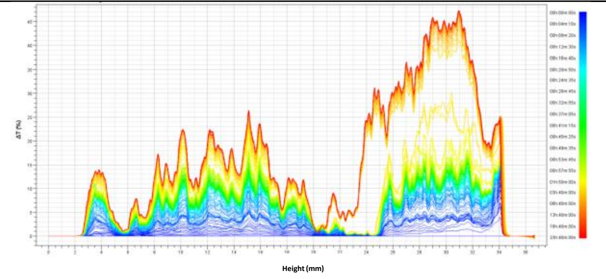	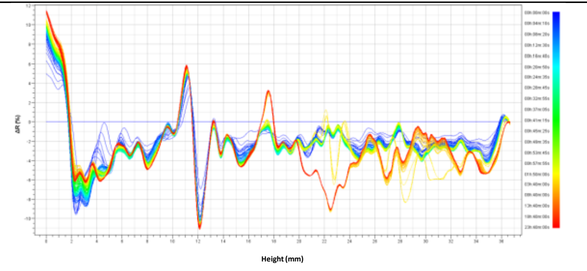
BNO-g-PMMA	
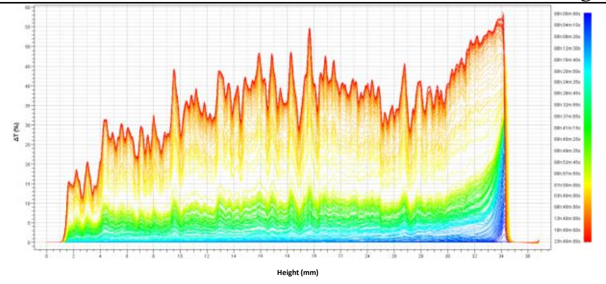	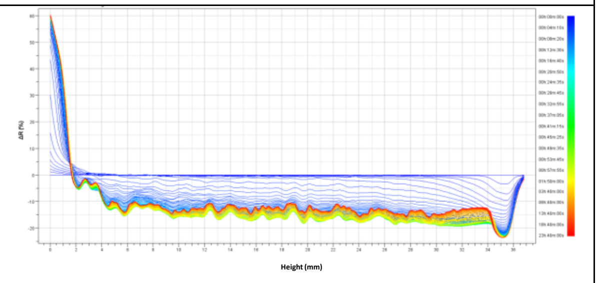
BNO-g-PS	

Figure S5. Multiple light scattering results for h-BN and silane-grafted h-BN in toluene.

The transmittance curve represents the transparence of the sample: the higher the amplitude of the transmitted light is, the more transparent the tube will be. The tube will be transparent if particles are small and few in number. The amplitude of transmitted light was really high generally around one peak. This peak was situated in the higher part of the tube, where particles were less numerous and small. This could be explained by the fact that transmitted light could not pass in the lower part of the tube due to the phenomenon of sedimentation, in which numerous very large particles accumulate mostly at the bottom and in the middle of the tube, blocking the transmission of light.

The backscattered curve represents the opacity of the sample: the higher the amplitude of the backscattered light is, the more opaque the tube will be. The tube will be opaque if there are numerous very large particles in suspension. Significant sedimentation was obvious only a few minutes after starting the experiments. The particles were compacted at the bottom of the vial, explaining why we observed a high, thin peak at the beginning of the curve. In the rest of the tube, particles were less numerous, and they continued to sediment as shown by a decreasing amplitude.

For the BNO-C18 in water, from the position h=30mm to the last position, the curve was stable, exactly like its transmittance curve. No light was transmitted or backscattered: the particles did not move. For the BNO-PS in water, a supernatant phase was seen: the particles accumulated at the top of the tube.

Figure S6 shows the grafting of BNO^{BM} with Si-C18. Ball milled hydroxylated particles BNO^{BM} were grafted with a Si-C18 silane agent. The FTIR (Figure S6a) spectrum of

BNO^{BM}/Si-C18 exhibited new bands at 2854 and 2926 cm^{-1} that corresponded to methyl groups of the alkyl part of the silane-grafting agent that was introduced at the edges of the h-BN platelets. The TGA (Figure S6b) showed a weight loss of almost 2 wt% attributed to the departure of the Si-C18 agent that was grafted. Pyrolysis, coupled with gas chromatography and mass spectrometry detection (Py-GC/MS), confirmed that the loss observed in TGA corresponded to the degradation of the C18 alkyl groups. The samples were pyrolyzed at 600 °C (Figure S6c). The chromatogram for h-BN showed no formation of organic compounds, while the BNO^{BM}/Si-C18 chromatogram showed several signals attributed to linear hydrocarbon molecules generally observed for the decomposition of long hydrocarbon chains. Indeed, this series of peaks corresponding to the alkane molecules of different lengths is an easily recognizable fingerprint that proved the presence of the octadecyl group of the Si-C18 agent. The characteristic peaks of aromatic compounds (benzene, toluene, styrene, etc.) were also present on the chromatogram, which were attributed to the thermal decomposition of this organic part of BNO^{BM}/Si-C18.

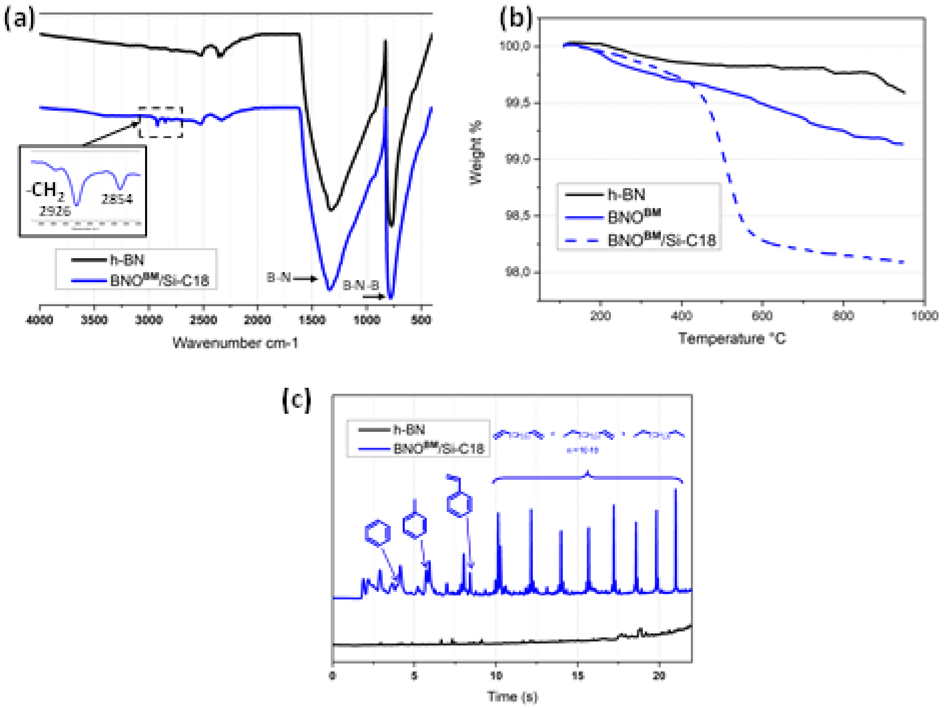

Figure S6. Grafting of BNO^{BM} with Si-C18 (**a**) FTIR spectra, (**b**) TGA graph, and (**c**) Py-GC/MS chromatograms of h-BN and BNO^{BM}/Si-C18.

References

73. Coleman, J.N.; Lotya, M.; O'Neill, A.; Bergin, S.D.; King, P.J.; Khan, U.; Young, K.; Gaucher, A.; De, S.; Smith, R.J.; et al. Two-Dimensional Nanosheets Produced by Liquid Exfoliation of Layered Materials. *Science* **2011**, *331*, 568–571, doi:10.1126/science.1194975.
